# Differential Effects of Infant Vocalizations on Approach-Avoidance Postural Movements in Mothers

**DOI:** 10.3389/fpsyg.2019.01378

**Published:** 2019-06-12

**Authors:** Daiki Hiraoka, Yuuki Ooishi, Ryoko Mugitani, Michio Nomura

**Affiliations:** ^1^Department of Education, Kyoto University, Kyoto, Japan; ^2^Japan Society for the Promotion of Science, Tokyo, Japan; ^3^NTT Communication Science Laboratories, Nippon Telegraph and Telephone Corporation, Kanagawa, Japan; ^4^The Faculty of Integrated Arts and Social Sciences, Japan Women’s University, Kanagawa, Japan

**Keywords:** parenting, infant vocalization, approach-avoidance behavior, emotional stimuli, Wii balance board

## Abstract

Infant vocalization plays a pivotal role in communicating infant mood to parents and thereby motivating parenting responses. Although many psychological and neural responses to infant vocalization have been reported, few studies have examined maternal approach-avoidance behavior in response to infant vocalization. Thus, this research sought to determine how infant emotional vocalization affects maternal behavior. Twenty mothers participated in this behavioral study, all of whom had infants of 24 months old or less. In the experiment, they stood on a Balance Board that collected real-time data regarding center of pressure (COP), while listening to a series of infant vocalizations including cry, laugh, and babbling. They then listened to the same vocalizations for a second time and rated their felt emotions in response to each vocalization. The participants demonstrated significant postural movements of approaching in response to cry stimuli or to stimuli regarded as highly urgent. In contrast, they demonstrated postural movement of avoidance in response to laugh vocalization. These findings suggest that parenting behavior in response to infant emotional vocalization is regulated not by the pleasant-unpleasant axis but by the urgency of the stimulus.

## Introduction

Since human beings are born in a state of immaturity, human infant survival is highly dependent on the quality of parenting provided by their caregivers. Infants use various cues, including olfactory, somatosensory, visual, and auditory signals, to maintain maternal attention and induce response behavior. Among these cues, infants’ auditory cues are crucial for distal communication and signal the need for caregiving behavior in both non-humans and humans (for a review, Lonstein et al., 2015). Infant vocalization, particularly crying, communicates infants’ hunger or discomfort to caregivers, who are usually motivated to provide care in response (Soltis, 2004; Hiraoka and Nomura, 2016). This research focuses on the influence of infant vocalization and attempts to demonstrate the relation between maternal behavior and infant vocalization.

Many previous studies have examined mothers’ psychological, physiological, and neural responses to infant vocalization including laughing, crying, and babbling (e.g., Kim et al., 2016; Parsons et al., 2017c; Swain and Ho, 2017; Young et al., 2017). These previous studies have suggested that infant vocalization has the potential to trigger immediate and intuitive caregiving behavior from mothers based on a culturally common neural mechanism (Bornstein et al., 2017; Parsons et al., 2017c; Young et al., 2017). However, it should be also noted that human behavior in response to emotional stimuli is not unidirectional and may include both approach and avoidance responses (Gray, 1982; Berkman et al., 2009; De Carli et al., 2017). For example, humans are motivated to approach in response to pleasant stimuli and to avoid in response to aversive stimuli (Darwin, 1872; Gray, 1982; Hillman et al., 2004; Eerland et al., 2012; Brunyé et al., 2013). Infant laughing has been found to elicit mothers’ pleasure and reward systems (Seifritz et al., 2003; Kringelbach et al., 2016), while infant crying elicits psychological aversiveness and stress in mothers (Reijneveld et al., 2004; Talvik et al., 2008; Fujiwara et al., 2011). This would suggest that infant laughing and crying would induce mothers’ approach and avoidance behavior, respectively. However, another possibility should be noted that infant cry vocalizations may elicit mothers’ approach behavior since early infant crying is an important means by which infants can maintain contact with the mother (Bell and Ainsworth, 1972).

Christensson et al. (1995) revealed that infants reliably cry when separated from their mothers and stop crying at reunion. Additionally, as infant crying particularly triggers motor evoked potentials in parents that is considered as a rapid motor preparation for caregiving behavior (Messina et al., 2016), despite the aversiveness, mothers may be motivated to engage in approach behavior in response to crying infants to contact and provide care for them. Lange-Küttner (2010) showed that high frequency sound (e.g., garden bird song) attracts adult attention more than low frequency sound (e.g., sea bird sound). Infant crying, which is a higher frequency voice than other infant vocalizations, is considered a signal that elicits parental attention and approach behavior (Bell and Ainsworth, 1972; Soltis, 2004). However, an avoidance response toward infants’ sad expressions has recently been demonstrated by De Carli et al. (2017). Although the data in their research are of great value in providing a preliminary investigation of the approach-avoidance response to infant stimuli, their sample was made up of only university students and some previous research has revealed that mothers exhibit specific responses to infant stimuli compared to non-parents (Rigo et al., 2019). These authors showed that the activation/deactivation pattern of default mode network in response to infant crying differs between mothers and nulliparous women. It suggests that caregivers would be specifically attracted and motivated to take care by infant crying stimuli compared to non-caregivers. Therefore, in this study, we recruited mothers and aimed to investigate maternal approach-avoidance behavior in response to infant stimuli.

The objective of this study was to investigate the effects of the emotional value of infant vocalization on maternal approach-avoidance behavior and further, to evaluate the relationship between maternal behavior and their felt emotions.

We also investigated the relation between maternal approach-avoidance behavior and subjective felt emotion in response to infant vocalization. Brunyé et al. (2013) proposed that an individualized approach should be adopted to understand the relationship between emotion and behavior because the value of sensory stimuli is highly subjective. Evaluations of infant vocalization are highly subjective and various individual differences occur (for a review, Zeifman and St James-Roberts, 2017). Considering this, we assume that it is important to examine the relationship between the rating of infant vocalization stimuli and mothers’ actual behavior. The two primary aims of this study are as follows: (1) To investigate whether infant laugh stimuli (or stimuli rated as highly pleasant) would elicit maternal approach behavior, and (2) To verify whether infant cry stimuli (or stimuli rated as highly aversive) would elicit maternal avoidance behavior.

## Materials and Methods

### Participants

Based on the sample size used in previous research (Eerland et al., 2012), we recruited twenty-one mothers who participated in the experiment but one was excluded from the final analysis due to the failure of posture recording; consequently, we analyzed the data of 20 mothers. The average age of participants was 35.45 (Mean) ± 3.32 (SD) years old. All of them had reared infants, the average age of whom was 10.90 (Mean) ± 7.15 (SD) months old. Eight of the mothers (40%) had reared their first child, 10 (50%) had reared their second child, and 2 (10%) had reared their third child. All methods employed in this study were approved by the Ethics and Safety Committees of NTT Communication Science Laboratories, and were in accordance with the Declaration of Helsinki. All participants gave their informed consent, which was approved by the Ethics Committee of NTT Communication Science Laboratories. Mothers were paid 5,000 Yen (about 40 Euros) for their participation in the study.

### Experimental Tasks and Procedure

On the day of the experiment, participants were given general information about the experiment on arrival and their written consent was obtained. The experimental procedure consisted of two tasks. In the first task, participants’ postural sways were measured during presentation of infant vocalizations. In the second task, participants were asked to rate their subjective feelings in response to each infant vocalization. The same vocalization stimuli were used in both tasks.

#### Posture Task

Much of the previous research examining approach-avoidance behavior in response to emotional stimuli has employed approach-avoidance tasks using arm flexions and extensions or pulling, or pushing a joystick. These paradigms, however, carry the potential disadvantage that congruency effects are critically dependent on subjects’ cognitive interpretation of the task, which may reduce the ecological validity of the study (Stins et al., 2011). Given that arm movements or joystick manipulation are ambiguous in terms of approach and avoidance, instead, the current study employed an approach-avoidance task (Eerland et al., 2012; Brunyé et al., 2013) using a force plate to measure mothers’ postural sway. In this paradigm, the center of pressure (COP) was collected during presentation of stimuli. We used a balance Wii board with four weight sensors embedded in the board to calculate the COP. Participants were asked to stand on the board and forward movement of COP was defined as approach while backward movement was defined as avoidance. Some previous research using this paradigm has revealed that people move forward in response to pleasant pictures and backward in response to unpleasant pictures (Eerland et al., 2012; Brunyé et al., 2013).

The first 10 min of the posture task period were allocated for practice to familiarize participants with the experimental setup. Participants were asked to get on the Wii balance board and listen to three sample voice stimuli for practice. Wii balance board collected the COP data at 40 Hz to evaluate participants’ anterior-posterior sway After the practice, they listened to 60 experimental voice stimuli while standing on the Wii balance board. The order of the experimental voice stimuli was completely randomized for each participant; the intertrial interval was 9–15 s, and the duration for each stimulus was 6 s. After each 10 stimuli, participants got off the board and sat on a chair to rest for 1 min.

The Wii balance board has been confirmed as a valid and reliable measuring instrument for assessing standing balance and postural sway as professional grade force platforms (Clark et al., 2018). Wii balance board has also been used in previous studies to investigate postural sway in response to emotional stimuli (Eerland et al., 2012; Brunyé et al., 2013).

Eerland et al. (2012) argued that the effects of emotional stimuli on COP are expected to occur immediately and revealed that the valence of a picture affects COP movement within 1 s of the presentation of the stimulus. Parsons et al. (2017c) and Young et al. (2017) also indicated that infant vocalization is rapidly processed. In accordance with these studies, we divided the time series of COP data into 12 bins with a 500-ms window in each trial.

#### Subjective Rating Task

After completing the posture task, participants were asked to rate their felt characteristics for each infants’ voice stimulus (not aroused – aroused, displeased – pleased, not urgent – urgent, and healthy – sick) using a visual analog scale (VAS) ranging from 0 to 1 (Out et al., 2010, 2012; Joosen et al., 2013). VAS was presented on a PC screen using PsychoPy2 1.82 (Peirce, 2009). The length of the VAS line segment was about 20 cm and participants placed the cursor over the VAS, which was ranged from 0 to 1. According to the position, PsychoPy2 calculated the value to the nearest one one-hundredth. To evaluate the degree of participants’ intention to approach or avoid the voice stimuli, we also asked them to rate their desire to pick up the baby (pick up) or leave the baby alone (harsh) using a VAS.

#### Infants’ Voice Stimuli

**FIGURE 1:**
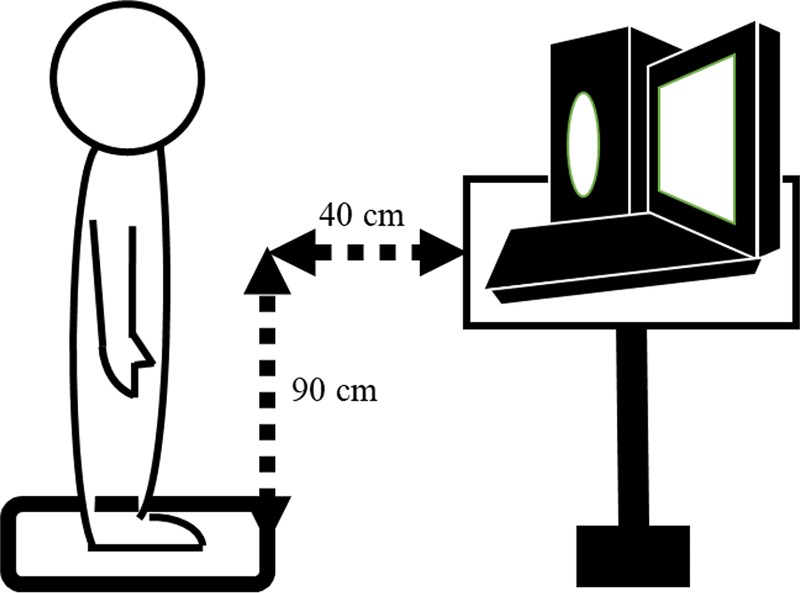
Setting of posture task.

In total, we used 60 infants’ voice stimuli, consisting of 20 infant cry stimuli, twenty infant laugh stimuli, and twenty infant babble stimuli, all of which were collected from the NTT Infant Speech Database (Amano et al., 2008). This database was compiled from longitudinal (up to 5 years) recordings of spontaneous vocalizations of five infants and their parents. We selected the experimental voice stimuli from 3- to 12-month-old infants’ vocalizations. The 3–12 month period corresponds to the Expansion stage and the Canonical stage in infant speech expression development (Oller, 2000). Infant laughter appears in this stage at about 4 months (Oller, 2000). Following this period is the Integrative stage where infant vocalization adopts linguistic characteristics. In order to examine the effect of vocalization on maternal response without verbal information, we used infant vocalizations as stimuli. In this database, each voice is labeled as one of either laugh, cry, or babble. In the current study, the three experimenters re-labeled all the voices and then used vocalizations that were unanimously labeled the same by all three experimenters. The sound pressure levels (SPL) of all infants’ voice stimuli were adjusted so that they did not exceed an A-weighted SPL of 70 dB in the fast mode. The loudspeaker was positioned just to the left side of the laptop, in front of participants at a distance of 40 cm and a height of 90 cm (see Figure 1).

### Data Analysis and Statistical Evaluation

**FIGURE 2:**
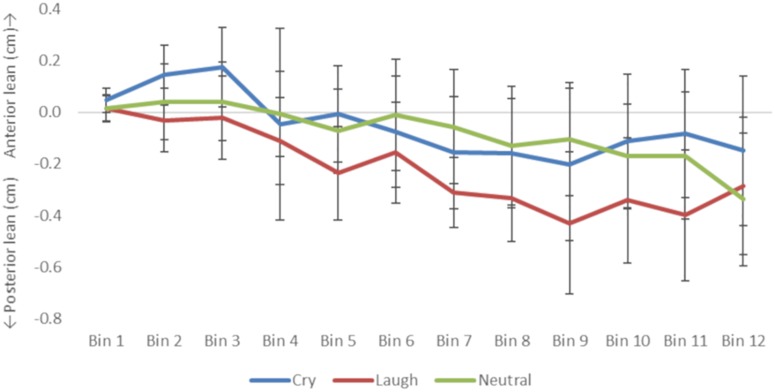
Time-binned mean and Confidence Interval of center of pressure (COP) in cm along the anterior–posterior axis for each of the three vocalization categories.

First, participants’ ratings of subjective feelings in response to the sounds were examined using One-Way repeated measures ANOVAs. Differences between the stimulus categories were examined using paired samples *t*-tests with the Holm method. Then, to examine the effect of infant vocalizations on maternal postural sway, the first value between 240 data points of COP for each infant vocal stimulus was set to 0 cm (baseline) as in previous research (Bakdash and Marusich, 2017), and we averaged the data with a 0.5-s window (*t* = 0 to 0.5, *t* = 0.5 to 1.0, *t* = 1.0 to 1.5, and *t* = 5.5 to 6.0 for each). These 12 averaged COPs were defined as A1–A12, and the corresponding periods were defined as Bin 1–Bin 12, respectively. The time series data of COP were expressed as means ± 95% Confident Interval (cm) (Table 2 and Figure 2). Time *t* = 0 was set as the time of the voice-stimulus onset. The difference between the averaged COP was analyzed with a two-way repeated measures ANOVA with type of vocal stimulus (laugh, cry, and babble) and time (baseline, Bin 1–Bin 12) as within-subjects factors. Then, the differences between the averaged COPs (A1–A12) and the baseline were analyzed using Dunnett’s method. The differences between the averaged COPs of vocal types were analyzed using Holm’s method.

We then performed a multilevel correlation analysis to estimate the relation between subjective feeling and each postural movement in Bin 1–12 in response to infant vocalizations. All analyses were conducted using R 3.1.2. Multiple comparison was conducted using the multcomp package (Hothorn et al., 2008) and multilevel correlation was conducted using the rmcorr package (Bakdash and Marusich, 2017), while a probability value of *p* < 0.05 was considered to be statistically significant.

## Results

### Descriptive Statistics

#### Effects of Parenting Experience on Each Variable

We used independent-samples *t* tests to evaluate differences between primiparous and multiparous mothers in subjective ratings and the change in COP. There were no significant differences between primiparous and multiparous mothers (Arousal: *t* (18) = 1.12, *p* = 0.25; Valence: *t* (18) = 0.21, *p* = 0.84; Urgency: *t* (18) = 0.42, *p* = 0.68; Healthy: *t* (18) = 0.63, *p* = 0.28; Pick up: *t* (18) = 0.01, *p* = 0.99; Harsh: *t* (18) = 0.63, *p* = 0.54; and COP: *t* (18) = 0.04, *p* = 0.97).

#### Subjective Ratings for Each Infant Vocal Category

Table 1 shows the means and 95% confident intervals for each vocal category, and results of repeated measures of one-way ANOVA with type of vocal stimulus. For arousal, infant cry was rated as having higher arousal levels than infant laugh and neutral (cry vs. laugh: *t* (38) = 6.08, *p* < 0.01; cry vs. neutral; *t* (38) = 11.62, *p* < 0.01). It was also demonstrated that infant laugh was rated as higher in arousal than infant neutral (*t* (38) = 5.53, *p* < 0.01).

**Table 1:** Subjective ratings across the three vocalization categories.

	Cry			Laugh			Neutral							
	Mean	95% CI		Mean	95% CI		Mean	95% CI		*F*	*df*		*p*	*partial* η^2^
Arousal	0.81	0.77	0.86	0.64	0.60	0.68	0.48	0.44	0.52	67.57	2	38	<0.01	0.78
Valence	0.36	0.31	0.41	0.84	0.79	0.90	0.73	0.68	0.79	96.20	2	38	<0.01	0.84
Urgency	0.74	0.70	0.78	0.10	0.06	0.14	0.13	0.09	0.17	326.32	2	38	<0.01	0.94
Healthy	0.48	0.43	0.54	0.90	0.85	0.96	0.86	0.81	0.92	69.65	2	38	<0.01	0.79
Pick up	0.84	0.79	0.89	0.58	0.53	0.63	0.48	0.43	0.52	60.19	2	38	<0.01	0.76
Harsh	0.14	0.07	0.20	0.41	0.34	0.47	0.46	0.40	0.53	31.41	2	38	<0.01	0.62

For the ratings of the valence, infant cry was rated more negatively than infant laugh and neutral (cry vs. laugh: *t* (38) = 13.21, *p* < 0.01; cry vs. neutral; *t* (38) = 10.26, *p* < 0.01). It was also demonstrated that infant laugh received more positive ratings of valence than infant neutral (*t* (38) = 2.95, *p* < 0.01).

For ratings of urgency, infant cry was rated more urgent than infant laugh and neutral (cry vs. laugh: *t* (38) = 22.59, *p* < 0.01; cry vs. neutral; *t* (38) = 21.62, *p* < 0.01). Infant laugh and neutral stimuli were rated, similarly in terms of their urgency levels (*t* (38) = 0.96, *p* = 0.98).

For the health of the sounds, infant cry was rated as more healthy than infant laugh and neutral (cry vs. laugh: *t* (38) = 10.68, *p* < 0.01; cry vs. neutral; *t* (38) = 9.69, *p* < 0.01). Infant laugh and neutral stimuli were rated, similarly in terms of their health levels (*t* (38) = 0.99, *p* = 0.96).

For motivation to pick up the baby, infant cry elicited higher motivation to pick up than infant laugh and neutral sounds (cry vs. laugh: *t* (38) = 7.68, *p* < 0.01; cry vs. neutral; *t* (38) = 10.65, *p* < 0.01). Infant laugh elicited more motivation to pick up than infant neutral sounds (*t* (38) = 3.06, *p* < 0.01).

Finally, harsh ratings, infant cry elicited lower harsh ratings than infant laugh and neutral stimuli (cry vs. laugh: *t* (38) = 6.10, *p* < 0.01; cry vs. neutral; *t* (38) = 7.43, *p* < 0.01). Motivations of harsh parenting were rated, similarly for infant laugh and neutral stimuli (*t* (38) = 1.33, *p* = 0.57).

### Postural Sway Analyses for Each Infant Vocal Category

Table 2 shows the mean COP and 95% confidence interval. The first COP was corrected to zero in each trial. Therefore, all COP values represent the amount of change from the onset of sound presentation. To address the time series variation of COP, the 6000 ms time was collapsed to 12 periods (500 ms each; Brunyé et al., 2013); data from each time Bin are detailed in Table 3 and Figure 2.

**Table 2:** Center of pressure (COP) movement (cm) in the anterior–posterior direction across the three vocalization categories.

Vocalization	Mean	95% CI lower	95% CI upper
Cry	0.00	−0.17	0.17
Neutral	−0.07	−0.24	0.09
Laugh	−0.24	−0.41	−0.07

**Table 3:** Time-binned mean and Confidence Interval of COP in cm along the anterior–posterior axis for each of the three vocalization categories.

		Bin 1	Bin 2	Bin 3	Bin 4	Bin 5	Bin 6	Bin 7	Bin 8	Bin 9	Bin 10	Bin 11	Bin 12
Cry	Mean	0.05	0.14	0.18	−0.05	−0.07	−0.16	−0.16	−0.16	−0.20	−0.11	−0.08	−0.15
	95% CI	[−0.001, 0.14]	[0.03, 0.26]	[0.02, 0.33]	[−0.42, 0.33]	[−0.19, 0,25]	[−0.24, 0.20]	[−0.38, 0.05]	[−0.36, 0.12]	[−0.33, 0.16]	[−0.35, 0.13]	[−0.36, 0.14]	[−0.41, 0.11]
Laugh	Mean	0.01	−0.02	0.00	−0.10	−0.17	−0.20	−0.33	−0.38	−0.46	−0.40	−0.40	−0.44
	95% CI	[−0.05, 0.08]	[−0.18, 0.15]	[−0.20, 0.19]	[−0.30, −0.10]	[−0.39, 0.05]	[−0.42, 0.02]	[−0.54, −0.11]	[−0.62, −0.14]	[−0.71, −0.21]	[−0.64, −0.16]	[−0.65, −0.15]	[−0.70, −0.18]
Neutral	Mean	0.03	0.16	0.06	−0.04	0.07	0.03	−0.15	−0.21	−0.13	−0.18	−0.22	−0.29
	95% CI	[−0.03, 0.10]	[−0.10, 0.32]	[−0.14, 0.25]	[−0.24, 0.16]	[0.15, 0.29]	[−0.19, 0.25]	[−0.37, 0.07]	[−0.45, 0.03]	[−0.38, 0.12]	[−0.42, 0.06]	[−0.47, 0.03]	[−0.55, −0.02]

#### Difference in the Change in COP for Infant Vocal Categories

To compare the difference in effect of the category of infant vocalization on maternal postural sway, we conducted a three (sound category) × thirteen (time) repeated measures ANOVA. This analysis revealed a significant main effect of time, *F* (12, 228) = 5.56, *p* = 0.002, *partial* η^2^ = 0.22. Dunnett’s method demonstrated that COP was significantly less than 0 at bin 9 (*t* (228) = 3.43, *p* = 0.04) and bin 12 (*t* (228) = 3.60, *p* = 0.03). The ANOVA also revealed a marginal main effect for sound category, *F* (2, 38) = 2.73, *p* = 0.078, η^2^ = 0.13. Holm’s method multiple comparison demonstrated that infants’ cry stimuli marginally elicited participants’ forward sway movement compared to laugh stimuli (*t* (38) = 2.18, *p* = 0.10). The interaction of time and sound categories was not significant, *F* (22, 418) = 1.15, *p* = 0.337, *partial* η^2^ = 0.06. The ANOVA was performed by applying the COP value with the maximum value of the variation for each trial corrected to one as a dependent variable. Similar results to the analysis above were obtained (main effect of time: *F* (12, 228 = 6.55, *p* < 0.001, *partial* η^2^ = 0.26; main effect of sound category: *F* (2, 38) = 2.81, *p* = 0.08, *partial* η^2^ = 0.06; interaction: *F* (22, 418) = 1.18, *p* = 0.316, *partial* η^2^ = 0.06).

### Multilevel Correlation

To investigate the relation between maternal postural movement and subjective feeling for infant vocalization, we performed a multilevel correlation analysis between the COP of each Bin and each subjective rating. The results of multilevel correlation analysis are presented in Table 4. We found a significant positive correlation between COP and perceived urgency at Bins 1–3 (Bin 1: *r* = 0.07, *p* = 0.02; Bin 1: *r* = 0.07, *p* = 0.02; and Bin 3: *r* = 0.06, *p* = 0.046). There was also a significant negative correlation between COP and perceived pleasantness at Bin 1 (*r* = −0.06, *p* = 0.04). These results suggest that participants immediately approach vocalizations that they perceive to be either urgent or unpleasant. Moreover, we tested the correlation between COP and the subjective rating in each sound category. There was no significant correlation (Table 5).

**Table 4:** Multilevel correlation between COP in cm along the anterior–posterior axis and subjective ratings (0–6000 ms).

	Arousal	Valence		Urgency		Healthy	Pick up	Harsh
Bin 1	0.04	−0.06	^∗^	0.07	^∗^	−0.05	0.03	−0.03
Bin 2	0.02	−0.05		0.07	^∗^	−0.03	0.03	−0.03
Bin 3	0.02	−0.04		0.06	^∗^	−0.03	0.04	−0.02
Bin 4	0.02	−0.03		0.05	+	−0.03	0.03	−0.02
Bin 5	0.02	−0.03		0.04		−0.02	0.01	−0.03
Bin 6	0.00	−0.02		0.02		−0.01	0.00	−0.02
Bin 7	0.00	−0.02		0.01		−0.01	0.01	0.00
Bin 8	0.02	−0.04		0.03		−0.02	0.02	0.00
Bin 9	−0.01	−0.03		0.03		−0.02	0.01	−0.01
Bin 10	−0.01	−0.03		0.03		−0.01	0.02	−0.01
Bin 11	0.01	−0.04		0.04		−0.02	0.02	−0.01
Bin 12	0.01	−0.03		0.04		−0.02	0.01	−0.01

*^∗^p < 0.05, +p < 0.10.*

**Table 5:** Multilevel correlation between COP, measured in centimeters, along the anterior–posterior axis and subjective ratings in each sound category.

	COP					

	Arousal	Valence	Urgency	Healthy	Pick	Harsh
Cry	0.083	−0.063	0.059	0.037	0.014	−0.040
Neutral	0.054	0.009	0.067	0.000	0.099	−0.075
Laugh	−0.013	0.022	0.032	−0.027	−0.005	−0.033

## Discussion

As noted in the Introduction, the existing literature on how infant vocalizations affect maternal behavioral responses is relatively scarce. Thus, the present study was designed to determine the effect of infant emotional vocalizations on maternal approach-avoidance behavior. One interesting finding of our study is that vocalization perceived as urgent and unpleasant elicited maternal approach behavior in the early stage of vocal stimuli, with mothers marginally approaching crying vocalizations. A supplementary analysis also demonstrated that infant crying elicited forward movement in the early stage. These outcomes are contrary to some previous studies that have found that sad infant facial expressions elicit a tendency for avoidance (De Carli et al., 2017), and that pleasant emotional stimuli elicit approach behavior while unpleasant stimuli elicit avoidance behavior (Hillman et al., 2004; Eerland et al., 2012; Brunyé et al., 2013). These differences might be explained by the fact that our research recruited mothers of infants because previous research have revealed that mothers’ response to infant stimuli differ on both behavioral and neural level when compared to non-mothers (Thompson-Booth et al., 2014; Parsons et al., 2017b). Details and directions for future research are discussed below.

This study confirmed that vocalizations perceived as urgent trigger approach behavior rather than avoidant behavior. This is consistent with earlier studies, suggesting that the role of infant distress is to cue mothers to come into proximity to the baby (Bell and Ainsworth, 1972; Messina et al., 2016; Parsons et al., 2017a; Young et al., 2017). Although previous studies have also revealed that approach-avoidance behavior is associated with the valence of each item (Gray, 1982; Hillman et al., 2004; Eerland et al., 2012), our results suggest that mothers’ behavioral tendencies in response to infant emotion are regulated by a specific system based on perceived urgency. Our results extend the findings of Bornstein et al. (2017) and Parsons et al. (2017c) in demonstrating that intuitive parenting is rapidly processed with neural networks, including the subcortical and motor area, because we were able to demonstrate the rapid behavioral response of mothers to infant vocalization. The current finding is also consistent with previous results indicating that infant crying specifically elicits motor evoked potential for preparation to move (Messina et al., 2016). However, these results seem to be inconsistent with the recent study of De Carli et al. (2017) in demonstrating maternal avoidance response toward infants’ sad expressions. This result may be derived from the fact that, in our study, it was mothers who participated in the experiments, while De Carli et al. (2017) recruited childless individuals regardless of the fact that their brain structure and function are sufficiently developed in the postpartum period to provide sensitive parenting (Kim et al., 2010; Parsons et al., 2017b). Mothers who are raising their infants are highly sensitive to the need of infants to provide maternal care to infants, and infant crying is a strong motivator for them to provide maternal care. In this study, mothers perceived cries as a signal of need in the infant and automatically moved toward the vocalization. A future study should be conducted to examine the difference between mothers and non-mothers under the same paradigm.

Contrary to expectations, this study did not find a significant mothers’ forward movement by infant laughter. A possible explanation for this might be that mothers felt infant laughter as pleasant but not appealing stimuli which elicit maternal approach behavior. Infant crying is an effective way of communicating infants’ physical pain and illness and of driving parents’ need to confirm the safety of the infant (Soltis, 2004). Infant laughing or babbling seems less urgent (Provine, 2004) and does not, therefore, immediately attract the mother’s attention or motivate her approach behavior. The current study demonstrated that mothers gradually moved backward throughout all infant vocalization categories. To the best of our knowledge, little research has investigated the effect of sound on approach-avoidance postural sway and it is possible that sound stimuli would commonly elicit gradual backward movement, partly because we used 70 dB sounds, which are very noisy. Future studies should address whether the gradual avoidance tendency in respect to sound stimuli is domain general or specific.

### Limitations and Future Directions

The absence of a significant overall effect for sound category must be acknowledged, and it is possible that we did not examine any individual difference in maternal responses to infant vocalizations, which would make it difficult to detect effects. De Carli et al. (2017) demonstrated that childhood adversity modulated the approach-avoidance response to infant emotional cues. While the current study is worthy from the viewpoint of the general tendency in maternal behavior, future studies should address individual differences in maternal approach-avoidance behavior to infant vocalization. Recently, several studies have revealed that Oxytocin receptor gene polymorphism moderates the effects of childhood experiences on behavioral and physiological responses to infant crying (Esposito et al., 2017; Hiraoka and Nomura, 2019; Senese et al., 2019). Since this gene x environment approach is useful for revealing the mechanism shaping the parenting behavior, future research should examine the gene x environment effects on maternal postural movement. The neural or endocrine systems underlying maternal approach-avoidance behavior in response to infant vocalization were also not addressed. It is possible that oxytocin, which is associated with sensitive parenting (Feldman, 2012), affects maternal behavioral response to infant vocalization. Furthermore, it would be valuable to address the relationship between maternal approach-avoidance behavior and maternal psychological health and child developmental outcome. McElwain and Booth-Laforce (2006) revealed that sensitive maternal response to a crying infant predicts secure attachment shaping; therefore, the approach behavior in the current result is likely to be associated with healthy child development. However, parents are shown to become unresponsive to infant stimuli as their children grow up (Parsons et al., 2017a). Highly sensitive parents could respond quickly to a child’s demand, a status that is not always desirable (Winnicott, 1956) and that would impose a great burden on the mother. Addressing the relationship between maternal immediate approach behavior and mother’s psychological health would contribute to the creation of a clinical tool for assessing or intervening in parenting.

## Conclusion

To the best of our knowledge, this is the first study to investigate the effect of infant vocalizations on maternal approach-avoidance behavior. We found that infant crying or vocalization perceived as urgent elicits immediate approach behavior by mothers. These results are different from the traditional consideration that connects approach behavior to pleasant stimuli and avoidance behavior to aversive stimuli and suggests that reactions to infant emotional signals are specific to mothers. The current paradigm addressing maternal COP movement and the findings may contribute to various research areas related to parenting and mother-infant interactions in theoretical way. For example, previous observations lack insight into the internal process of actual behaviors mothers take in response to infant vocalization, and neuroimaging studies have indicated maternal response to infant cue as indirect observations on a neural level. However, the current results of maternal postural movements in response to infant vocalizations might serve as a direct behavioral indicator of parental response to infant cue.

## Data Availability

The datasets generated for this study are available on request to the corresponding author.

## Ethics Statement

All methods employed in this study were approved by the Ethics and Safety Committees of NTT Communication Science Laboratories, and were in accordance with the Declaration of Helsinki. Before participation, all participants read the explanatory form describing the research aim, procedures, the way of data handling, and potential risks. Then, all participants gave their informed consent, which was approved by the Ethics Committee of NTT Communication Science Laboratories.

## Author Contributions

DH, YO, RM, and MN designed the current study. RM recruited the participants. DH, YO, and RM collected the data. DH and YO analyzed the data. DH, YO, RM, and MN interpreted the findings, and DH wrote the main manuscript. All authors reviewed the manuscript.

## Conflict of Interest Statement

The authors declare that the research was conducted in the absence of any commercial or financial relationships that could be construed as a potential conflict of interest.

## Supplementary Information

As noted in the main report, Table 3 shows the means and 95% confident intervals of COP for each vocal category. Although the interaction of repeated measures of two-way ANOVA with sound category and time was not statistically significant, some confidence intervals did not include zero, indicating that there were significant movements from the baseline position. In Bin 2 and Bin 3, COPs during presentation of infant crying were larger than zero (Bin 2: *t* (19) = 2.58, *p* = 0.01; and Bin 3: *t* (19) = 2.38, *p* = 0.03), which indicates that cry vocalization elicits anterior movement after stimulus onset and the movement continues for approximately 1500 ms. In contrast, infant laugh elicited posterior movement from Bin 7 to Bin 12 (Bin 7: *t* (19) = 4.84, *p* < 0.01; Bin 8: *t* (19) = 4.15, *p* < 0.01; Bin 9: *t* (19) = 3.26, *p* < 0.01; Bin 10: *t* (19) = 2.93, *p* = 0.01; Bin 11: *t* (19) = 3.31, *p* < 0.01; and Bin 12: *t* (19) = 2.23, *p* < 0.01). Infant neutral vocalization also elicited significant posterior movement at Bin 12 (Bin 12: *t* (19) = 2.75, *p* = 0.01).
